# Predictors of new-onset suicide risk and persistent suicide risk among young adult lesbian, gay and bisexual individuals experiencing the COVID-19 pandemic: a follow-up study

**DOI:** 10.1017/S2045796024000635

**Published:** 2024-11-04

**Authors:** Cheng-Fang Yen, Ching-Shu Tsai, Yu-Ping Chang, Peng-Wei Wang

**Affiliations:** 1 Department of Psychiatry, Kaohsiung Medical University Hospital, Kaohsiung, Taiwan; 2 Department of Psychiatry, School of Medicine, College of Medicine, Kaohsiung Medical University, Kaohsiung, Taiwan; 3 College of Professional Studies, National Pingtung University of Science and Technology, Pingtung, Taiwan; 4 Department of Child and Adolescent Psychiatry, Chang Gung Memorial Hospital, Kaohsiung Medical Center, Kaohsiung, Taiwan; 5 School of Medicine, Chang Gung University, Taoyuan, Taiwan; 6 School of Nursing, The State University of New York, University at Buffalo, New York, NY, USA

**Keywords:** anxiety, bisexual, depression, gay, internet, lesbian, psychological well-being, self-identity disturbance, sexual stigma, suicide

## Abstract

**Aims:**

The aim of this 4-year follow-up study was to examine the predictive effects of demographics, three types of sexual stigma, three types of self-identity confusion, anxiety, depression, family support and problematic Internet use before the coronavirus disease 2019 (COVID-19) pandemic on new-onset suicide risk and persistent suicide risk in young adult lesbian, gay and bisexual individuals who experienced the COVID-19 pandemic in Taiwan.

**Methods:**

Baseline data were collected from 1,000 lesbian, gay and bisexual individuals in 2018 and 2019. Outcome data on suicide risk were collected again in 2023. The suicide module of the Mini International Neuropsychiatric Interview was used to assess suicide risk in terms of thoughts of death, desire to self-harm, thoughts of suicide, plans for suicide and suicide attempts in the preceding month at the initial and follow-up assessments. Baseline three types of sexual stigma, self-identity disturbance, depression, anxiety and problematic Internet use were used to examine their prediction of new-onset suicide risk and persistent suicide risk at follow-up.

**Results:**

In total, 673 individuals participated in the follow-up survey. Notably, 16.5% of the participants who had no suicide risk at baseline had new-onset suicide risk at follow-up; 46.4% of the participants who had suicide risk at baseline also had suicide risk at follow-up. Participants who were transgender (*p* = .003), who perceived greater levels of microaggression (*p* < .001), and who had greater levels of problematic Internet use at baseline (*p* = .024) were more likely to have new-onset suicide risk at follow-up. Participants who had greater levels of self-identity confusion were more likely to have persistent suicide risk at follow-up (*p* = .023).

**Conclusion:**

Intervention strategies for reducing suicide risk in lesbian, gay and bisexual individuals should be developed with consideration of the predictors identified in this study.

## Introduction

The coronavirus disease 2019 (COVID-19) pandemic affected the health and lifestyle of many individuals. According to the World Health Organization, by 14 July 2023, more than 760 million individuals had contracted COVID-19, and nearly 7 million individuals had died from COVID-19 infection (World Health Organization [WHO], 2023a). Survivors of COVID-19 face the risk of COVID-19-related sequelae, such as fatigue and cognitive problems (Chen *et al.*, 2022). The COVID-19 pandemic negatively affected the accessibility and structure of education, the availability and cost of food, the economic activities, the mental health and the quality of life for people around the world (Onyeaka *et al.*, 2021). Marginalized individuals, including those of racial and ethnic minorities and of different genders, sexual orientations, health conditions, and socio-economic capabilities, were disproportionately affected by the COVID-19 pandemic relative to the general population.Lesbian, gay and bisexual (LGB) individuals were disproportionately affected by the COVID-19 pandemic in terms of mental health compared with heterosexual individuals (Buspavanich *et al.*, 2021; Slemon *et al.*, 2022; Veldhuis *et al.*, 2020). A meta-analysis that included 15 articles investigating the mental health of LGB individuals during the COVID-19 pandemic revealed pooled prevalence rates of 58.6%, 57.6% and 52.7% for anxiety, depression and psychological distress, respectively, for such individuals (Batra *et al.*, 2023). The effects of the COVID-19 pandemic and of pandemic-prevention measures on the mental health of LGB individuals warrant investigation.

Before the COVID-19 pandemic, young LGB individuals were at higher risk of engaging in suicidal behaviours than were heterosexual individuals (Miranda-Mendizábal *et al.*, 2017). A meta-analysis that included 44 articles found that LGB bias-based victimization, bullying and negative family treatment were significantly associated with suicidal ideation and suicide attempts among LGB individuals (de Lange *et al.*, 2022). The COVID-19 pandemic amplified prepandemic inequities related to stigma, finances, access to employment and healthcare among LGB individuals (Buspavanich *et al.*, 2021; Chen, 2022; Diaz *et al.*, 2021; Gibb *et al.*, 2020; Kamal *et al.*, 2021; Krause, 2021; Salerno *et al.*, 2020; Suen *et al.*, 2020). For example, sexual minority young people had significantly higher levels of depression and post-traumatic stress disorder symptoms as well as COVID-19-related worries and grief than heterosexual people (Kamal *et al.*, 2021). Compared to heterosexual older adults, sexual minority older adults had more emotional stress and were more likely to have changes in income and work during the pandemic (Chen, 2022). A meta-analysis revealed that 28.8% of LGB individuals reported feeling suicidal during the COVID-19 pandemic (Batra *et al.*, 2023). Identifying the factors that predict suicide risk among LGB individuals who experienced the COVID-19 pandemic is a key step in developing intervention strategies for addressing suicide.

Several problems related to the predictors of suicide risk among LGB individuals who experienced the COVID-19 pandemic have been identified. First, no prospective study has determined the rates of new-onset suicidal ideation (i.e., suicidal ideation detected at follow-up but not at baseline) and persistent suicidal ideation (i.e., suicidal ideation detected at baseline and at follow-up) in LGB individuals who experienced the COVID-19 pandemic. In the Taiwanese Study of Sexual Stigma (T-SSS), conducted in 2018 and 2019, data regarding suicidal ideation in 1,000 LGB individuals in emerging adulthood living in Taiwan were collected (Chen *et al.*, 2023, 2021; Hsieh *et al.*, 2021; Lee *et al.*, 2022; Lin *et al.*, 2022; Tsai *et al.*, 2021; Yen *et al.*, 2021). Comparing suicide risk in these participants before and after the COVID-19 pandemic can reveal the rates of new-onset and persistent suicidal ideation and attempt in LGB individuals who experienced the COVID-19 pandemic.

Second, according to ecological systems theory (Bronfenbrenner, 1979), multiple individual and environmental factors contribute to the incidence of suicidal ideation and attempt in LGB individuals. Several individual factors (e.g., depression symptoms, hopelessness, impulsivity and age of first same-sex attraction), environmental factors (e.g., low family support) and social factors (e.g., bullying and victimization) are predictors of suicide in LGB individuals (Liu and Mustanski, 2012; Mustanski and Liu, 2013; Tan *et al.*, 2021). Cross-sectional studies have also reported that individual factors (e.g., younger age and neuroticism), environmental factors (e.g., discomfort with family members and family conflict related to sexual orientation) and social factors (e.g., being discriminated against based on sexual orientation) were significantly associated with suicidal ideation or attempt in LGB individuals (Lozano-Verduzco *et al.*, 2023; Suen *et al.*, 2020). The T-SSS collected data on individual factors (sexual orientation, gender identity, self-identity confusion, depression, anxiety and problematic Internet use), environmental factors (perceived family support) and social factors (sexual stigma) from LGB individuals before the COVID-19 pandemic. Whether these multidimensional factors can predict new-onset suicide risk and persistent suicide risk among LGB individuals who experienced the COVID-19 pandemic warrants investigation.

Third, discrimination based on sexual orientation has been associated with suicide in studies conducted before the COVID-19 pandemic (Liu and Mustanski, 2012; Mustanski and Liu, 2013) and in a study conducted during the COVID-19 pandemic (Lozano-Verduzco *et al.*, 2023). The T-SSS collected data from LGB individuals on various aspects of their experiences of sexual stigma, including perceived familial sexual stigma, internalized sexual stigma and perceived sexual orientation microaggression. Whether different aspects of experiences of sexual stigma have different predictive effects on suicide risk in LGB individuals who experienced the COVID-19 pandemic warrants investigation. Fourth, transgender LGB individuals experience dual minority-related stress (Thoma *et al.*, 2021). Furthermore, problematic Internet use is more prevalent in LGB individuals than in heterosexual individuals (Broman *et al.*, 2018); problematic Internet use was significantly associated with depression and anxiety in LGB individuals (Huang *et al.*, 2022). Whether being transgender and having problematic Internet use predict suicide risk in LGB individuals who experienced the COVID-19 pandemic warrants further investigation.

The present study, which had a 4-year follow-up period, analyzed the predictive effects of individual, environmental and social factors on new-onset suicide risk and persistent suicide risk among young adult LGB individuals who experienced the COVID-19 pandemic. This study hypothesized that being transgender, perceiving sexual stigma (e.g., familial sexual stigma, internalized sexual stigma, sexual orientation microaggression) and having self-identity confusion (e.g., disturbed identity, unconsolidated identity, or lack of identity), depression, anxiety, problematic Internet use and low family support before the COVID-19 pandemic would predict new-onset suicide risk and persistent suicide risk among LGB individuals who experienced the COVID-19 pandemic.

## Methods

### Participants and procedure

Both the criteria and methodology used in the T-SSS for recruiting participants at baseline have been described in previous studies (Chen *et al.*, 2023, 2021; Hsieh *et al.*, 2021; Lee *et al.*, 2022; Lin *et al.*, 2022; Tsai *et al.*, 2021; Yen *et al.*, 2021). In brief, a cohort of 1,000 individuals (500 men and 500 women) at baseline were recruited through online advertisements on social media platforms – including Facebook, Twitter, and LINE – and a bulletin board system from August 2018 to June 2019. The inclusion criteria were identifying as an LGB individual, being 20–30 years of age, and living in Taiwan. The exclusion criterion was having any form of impaired cognition that might have interfered with the ability to complete a questionnaire.

Four years later, the same 1,000 individuals were contacted by text message and invited to participate in a follow-up study. Those who responded to this message and agreed to participate received a blank consent form, a research questionnaire and instructions for how to complete the questionnaire; they were also allowed to contact the research assistant for help if they had any problem understanding the questionnaire. A total of three text messages were sent to the potential participants to invite them to participate in the follow-up study, with a 1-month interval between each pair of messages. Those who responded to none of these messages were considered to have been lost to follow-up. This study was approved by the Institutional Review Board of KMUH (KMUHIRB-F(I)-20,210,219).

### Measures

#### Outcome variable: Suicide risk

Five items in the suicide module of the Mini International Neuropsychiatric Interview (Sheehan *et al.*, 1998) were used to assess suicide risk in terms of thoughts of death, desire to self-harm, thoughts of suicide, plans for suicide and suicide attempts in the preceding month at the initial and follow-up assessments. Participants answered “yes” or “no” to each item. Participants who answered “yes” to any of the items were considered to have suicide risk. Participants were divided into four groups according to suicide risk in the initial and follow-up assessments. Participants who had suicide risk at the follow-up but not at the initial assessment were categorized into the new-onset suicide risk group. Participants who did not have suicide risk at either of the assessments were categorized into the no suicide risk group. Participants who had suicide risk at both assessments were categorized into the persistent suicide risk group. Participants who had suicide risk at the initial but not at the follow-up assessment were categorized into the remitted suicide risk group.

#### Predicting variables at baseline

Participants’ demographics, three types of sexual stigma, self-identity confusion, depression, anxiety, problematic Internet use and perceived family support were measured at baseline.

#### Demographic, sexual and sex-related characteristics

We collected data regarding each participant’s sex, age, educational level (high school or lower level of education vs. college or higher level of education), sexual orientation (lesbian or gay vs. bisexual) and gender orientation (transgender or not).

#### Homosexuality-Related Stigma Scale (HRSS)

The 12-item HRSS was used to measure the participants’ levels of perceived sexual stigma from family members (Liu *et al.*, 2009). Each item was rated on a 4-point Likert scale with endpoints ranging from 1 (*strongly disagree*) to 4 (*strongly agree*). Scores for individual items were summed to obtain an overall HRSS score. A higher score indicated that the participant perceived a greater level of sexual stigma from family members (Liu *et al.*, 2009). The Cronbach’s α coefficient for this scale was .93 in this study.

#### Measure of Internalized Sexual Stigma for Lesbians and Gay Men (TC-MISS-LG)

The 17-item traditional Chinese version of the TC-MISS-LG was used to assess each participant’s sexuality, identity and level of social discomfort (Lingiardi *et al.*, 2012). Each item was rated on a 5-point Likert scale with endpoints ranging from 1 (*strongly disagree*) to 5 (*strongly agree*). A higher score indicated a greater degree of internalized sexual stigma. Psychometric evidence supports the reliability and validity of the TC-MISS-LG for the Taiwanese population (Yen *et al.*, 2021). The internal consistency values of the TC-MISS-LG were acceptable in the present study (Cronbach’s α = .76).

#### Sexual Orientation Microaggression Inventory (SOMI)

The 19-item traditional Chinese version (Hsieh *et al.*, 2021) of the SOMI (Swann *et al.*, 2016) was used to assess microaggression in the dimensions of anti-gay attitudes and expressions, denial of homosexuality and societal disapproval over 6 months among LGB individuals. Psychometric evaluation revealed a bifactor structure for the SOMI, with a general factor distinct from the other four trait factors (Swann *et al.*, 2016). Each item was rated on a 5-point Likert scale with endpoints ranging from 1 (*not at all*) to 5 (*almost every day*). A higher score indicated a greater level of microaggression. This version of the SOMI has acceptable internal consistency and concurrent validity (Hsieh *et al.*, 2021). The Cronbach’s α coefficient for this scale was .90 in the present study.

#### Self-Concept and Identity Measure (SCIM)

The traditional Chinese version of the 27-item SCIM was used to assess the level of current self-identity disturbance (Kaufman *et al.*, 2015, 2019). The SCIM assesses three dimensions of self-identity disturbance, including disturbed identity (for example, “Sometimes I pick another person and try to be just like them, even when I’m alone”), unconsolidated identity (for example, “When someone describes me, I am not sure if they are right or wrong”) and lack of identity (for example, “I feel like a puzzle and the pieces don’t fit together”). Items are rated on a 7-point rating scale ranging from 1 (strongly disagree) to 7 (strongly agree). A higher total score indicates a higher tendency for self-identity disturbance. The traditional Chinese version of the SCIM has acceptable congruent validity with bullying victimization (Wang *et al.*, 2019) and predictive validity for depression and suicidality 1 year later (Chen *et al.*, 2019). Cronbach’s α of the SCIM in the present study was .79.

#### Center for Epidemiologic Studies Depression Scale (CES-D)

The 20-item Mandarin Chinese version (Chin *et al.*, 2015) of the CES-D (Radloff, 1977) was used to assess the frequency of depressive symptoms within the preceding month. Each item was rated on a 4-point Likert scale with endpoints ranging from 1 (*rarely or none of the time*) to 4 (*most or all of the time*). A higher total score indicated more severe depression. The Cronbach’s α coefficient for this scale was .91 in this study.

#### State-Trait Anxiety Inventory (STAI)

The STAI consists of 20 items embedded in a single factor of anxiety. All the STAI items were assessed using a 4-point Likert scale, where a score of 1 indicates almost never and a score of 4 almost always. Therefore, a higher STAI score indicates greater anxiety (Spielberger *et al.*, 1983). Previous psychometric evidence showed that the STAI was reliable and valid for the Taiwan population (Lee *et al.*, 2017).

#### Family Adaptability, Partnership, Growth, Affection, and Resolve (APGAR) Index

The 5-item Chinese version (Chen *et al.*, 1980) of the APGAR Index (Smilkstein *et al.*, 1978) was used to assess the participants’ perceived support from their families. The Family APGAR Index was adapted to develop the Friend APGAR Index, which was used to assess the participants’ perceived support from their friends. Each item was rated on a 4-point Likert-type scale with endpoints ranging from 1 (*never*) to 4 (*always*). The total scores for the Family APGAR Index and Friend APGAR Index ranged from 5 to 20, with higher total scores for the Family APGAR Index and Friend APGAR Index indicating higher levels of perceived support from family and friends, respectively. In our study, the Cronbach’s α values for the Family APGAR Index and Friend APGAR Index were .94 and .93, respectively.

#### *Chen Internet Addiction Scale* (CIAS)

This study used the 26-item CIAS (Chen *et al.*, 2003) to assess the participants’ self-reported problematic Internet use severity in the month prior to completing the questionnaire. The participants rated each item on a 4-point scale ranging from 1 (*totally disagree*) to 4 (*totally agree*), with total scores ranging from 26 to 104. A higher total score indicated a higher level of problematic Internet use. The CIAS has acceptable reliability and validity among various populations in Taiwan (Chen *et al.*, 2003; Ko *et al.*, 2009). The Cronbach’s α coefficient of the CIAS in the present study was .92.

### Data analysis

All statistical analyses were conducted using SPSS 24.0 software (SPSS, Chicago, IL, USA). We employed descriptive statistics to summarize and analyze the participants’ demographic data, sexual stigma, self-identity confusion, depression, anxiety, perceived family function, problematic Internet use and suicide risk. The distributions of continuous variables were further tested for skewness and kurtosis to assess their level of departure from a normal distribution, and the results (i.e., absolute values of <3 for skewness and <10 for kurtosis) did not reveal any severe deviation (Lin *et al.*, 2013).

The individual associations of perceived sexual stigma (i.e., familial sexual stigma, internalized sexual stigma and sexual microaggression), self-identity confusion (i.e., disturbed identity, unconsolidated identity, and lack of identity), depression, anxiety, perceived family support and problematic Internet use at baseline with new-onset suicide risk (no suicide risk as the reference) and persistent suicide risk (remitted suicide risk as the reference) at follow-up were investigated using bivariable logistic regression analysis. Factors that were significantly associated with new-onset suicide risk and persistent suicide risk in bivariate logistic regression analysis were further entered into multivariable logistic regression analysis to determine their associations with new-onset suicide risk and persistent suicide risk. Forward conditional logistic regression analysis was used. Odds ratios (ORs) and 95% confidence intervals (CIs) were used to present the results of the analyses. Statistical significance was indicated by *p* < .05.

## Results

A total of 673 (67.3%) participants responded to the invitation and agreed to participate in the follow-up study, 167 (16.7%) responded to the invitation but refused to participate in the follow-up study and 160 (16.0) did not respond to the invitation. No differences in sex (χ^2^ = 0.005, *p* = .946), age (*t* = 1.890, *p* = .059), sexual orientation (χ^2^ = 2.087, *p* = .149) or suicide (χ^2^ = 0.834, *p* = .361) were observed between those who completed the follow-up survey and those who did not. Those who did not complete the follow-up survey were more likely to have an educational level of high school or lower (χ^2^ = 15.767, *p* < .001). All participants who self-identified as being transgender received the follow-up assessment.

Table 1 shows the demographics, sexual stigma, self-identity confusion, depression, anxiety, perceived family function and problematic Internet use in participants who received the follow-up assessment. The participants (*N* = 673) were nearly evenly distributed between two groups: men (50.1%) and women (49.9%). Their mean age was 24.8 years (standard deviation [SD] = 2.9 years) at baseline, and most of them had a college degree or higher (*n* = 618, 91.8%). More than half of them (*n* = 373, 55.4%) were gay or lesbian and 19 of them (2.8%) identified as transgender. The mean HRSS score was 26.8 (*SD* = 6.3), the mean TC-MISS-LG score was 35.6 (SD = 11.5), the mean SOMI score was 42.3 (SD = 11.3). The mean level of disturbed identity on the SCIM was 37.5 (SD = 9.7), unconsolidated identity was 29.4 (SD = 8.7), and lack of identity was 19.2 (SD = 7.5). The mean STAI score was 41.2 (SD = 12.7). The mean CES-D score was 18.9 (SD = 11.3). The mean APGAR Index score was 13.6 (SD = 3.6). The mean CAIS was 56.8 (SD = 15.3).

**Table 1.** Characteristics of participants who received the follow-up assessment (*N* = 673)

Variable	*n* (%)	Mean (SD)	Range
Gender			
Women	336 (49.9)		
Men	337 (50.1)		
Age at baseline (year)		24.8 (2.9)	20–30
Education level			
High school or below	55 (8.2)		
College or above	618 (91.8)		
Sexual orientation			
Bisexual	300 (44.6)		
Gay	373 (55.4)		
Transgender	19 (2.8)		
Perceived familial sexual stigma on the HRSS		26.8 (6.3)	10–40
Internalized sexual stigma on the MISS		35.6 (11.5)	17–76
Microaggression on the SOMI		42.3 (11.3)	19–78
Disturbed identity on the SCIM		37.5 (9.7)	13–67
Unconsolidated identity on the SCIM		29.4 (8.7)	10–64
Lack of identity on the SCIM		19.2 (7.5)	6–42
Anxiety on the STAI		41.2 (12.7)	20–79
Depression on the CES-D		18.9 (11.3)	0–57
Family support on the APGAR Index		13.6 (3.6)	5–20
Problematic Internet use on the CIAS		56.8 (15.3)	26–101

APGAR Index: Adaptability, Partnership, Growth, Affection, and Resolve Index; CES-D: Center for Epidemiologic Studies Depression Scale; CIAS: Chen Internet Addiction Scale; HRSS: Homosexuality-Related Stigma Scale; MISS: Measure of Internalized Sexual Stigma for Lesbians and Gay Men; SOMI: Sexual Orientation Microaggression Inventory; STAI: State-Trait Anxiety Inventory; SCIM: Self-Concept and Identity Measure.

**Figure 1.**
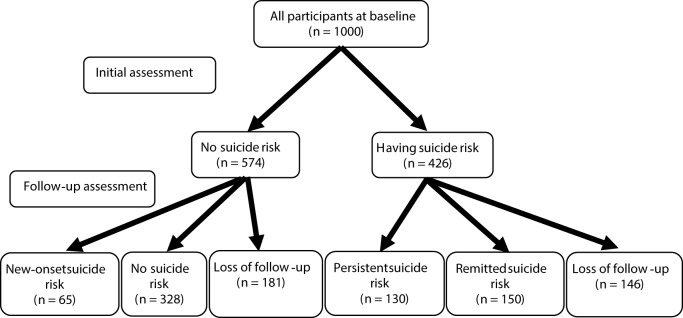
Suicide risk in initial and follow-up assessments.

The changes in suicide risk between the initial and follow-up assessments are illustrated in Fig. 1. At the initial assessment, 574 (57.4%) participants had no suicide risk; of them, 393 (68.5%) completed the follow-up assessment. A total of 65 (16.5%) participants who did not have suicide risk at the initial assessment but had suicide risk at the follow-up assessment were categorized into the new-onset suicide risk group; 328 (83.5%) participants who did not have suicide risk at either of the assessments were categorized into the no suicide risk group. At the initial assessment, 426 (42.6%) participants had suicide risk; of them, 280 (65.7%) completed the follow-up assessment. In total, 150 (53.6%) participants who had suicide risk at the initial assessment but not at the follow-up assessment were categorized into the remitted suicide risk group; 130 (46.4%) participants who had suicide risk at both assessments were categorized into the persistent suicide risk group.

The associations of perceived sexual stigma, self-identity confusion, depression, anxiety, perceived family support and problematic Internet use with new-onset suicide risk (no suicide risk as the reference) and persistent suicide risk (remitted suicide risk as the reference) are presented in Table 2. Participants who were transgender (*p* = .026), who perceived higher levels of microaggression (*p* < .001) and who had greater levels of self-identity confusion (*p* = .013), depression (*p* = .001) and problematic Internet use at baseline (*p* = .002) were more likely to have new-onset suicide risk at follow-up. Participants who had greater levels of self-identity confusion (*p* = .023) and depression at baseline (*p* = .045) were more likely to have persistent suicide risk at follow-up.

**Table 2.** Associations of factors with new-onset and persistent suicide risk: Bivariable logistic regression analysis

Variable	New-onset suicide risk^a^		Persistent suicide risk^b^	
	OR (95% CI)	*p*	OR (95% CI)	*p*
Gender^c^	0.958 (0.563–1.631)	.874	1.122 (0.701–1.795)	.632
Age	1.036 (0.945–1.136)	.452	0.984 (0.909–1.066)	.694
Education level^d^	0.742 (0.289–1.900)	.533	0.854 (0.381–1.915)	.702
Sexual orientation^e^	0.806 (0.472–1.376)	.429	1.345 (0.839–2.155)	.218
Transgender	3.821 (1.1741–2.440)	.026	7.210 (0.856–60.691)	.069
Perceived familial sexual stigma	1.025 (0.982–1.069)	.256	1.034 (0.994–1.075)	.101
Internalized sexual stigma	1.007 (0.983–1.030)	.578	0.994 (0.974–1.014)	.537
Microaggression	1.056 (1.030–1.082)	<.001	1.003 (0.983–1.022)	.780
Disturbed identity	1.026 (0.998–1.056)	.068	1.004 (0.980–1.029)	.735
Unconsolidated identity	1.019 (0.984–1.054)	.290	1.012 (0.987–1.038)	.353
Lack of identity	1.052 (1.011–1.095)	.013	1.037 (1.005–1.070)	.023
Anxiety	1.015 (0.992–1.039)	.209	1.015 (0.996–1.034)	.116
Depression	1.049 (1.021–1.079)	.001	1.022 (1.000–1.044)	.045
Family support	0.946 (0.874–1.023)	.162	0.944 (0.882–1.009)	.091
Problematic Internet use	1.028 (1.011–1.046)	.002	1.011 (0.995–1.028)	.175

CI: confidence interval; OR: odds ratio.

a The group of no suicide as the reference.

b The group of remitted suicide as the reference.

c Female as the reference.

d High school or below as the reference.

e Bisexual as the reference.

Factors that were significantly associated with new-onset suicide risk and persistent suicide risk were further entered in forward conditional logistic regression analysis models (Table 3). Participants who were transgender (*p* = .003), who perceived greater levels of microaggression (*p* < .001), and who had greater levels of problematic Internet use at baseline (*p* = .024) were more likely to have new-onset suicide risk at follow-up. Participants who had greater levels of self-identity confusion were more likely to have persistent suicide risk at follow-up (*p* = .023).

**Table 3.** Predictors of new-onset and persistent suicide risk: Forward conditional multivariable logistic regression analysis

Variable	New-onset suicide risk^a^		Persistent suicide risk^b^	
	aOR (95% CI)	*p*	aOR (95% CI)	*p*
Transgender	6.245 (1.836–21.244)	.003	–	–
Microaggression	1.054 (1.027–1.082)	<.001	–	–
Problematic Internet use	1.021 (1.003–1.039)	.024	–	–
Lack of identity	–	–	1.037 (1.005–1.070)	.023

CI: confidence interval; aOR: adjusted odds ratio.

a The group of no suicide as the reference.

b The group of remitted suicide as the reference.

## Discussion

Most of the participants (83.5%) who did not have suicide risk at baseline did not have suicide risk at follow-up either; however, 16.5% of the participants who did not have suicide risk at baseline did have suicide risk at follow-up. Furthermore, nearly half of the participants (46.4%) who had suicide risk at baseline still had suicide risk at follow-up. Asian LGB individuals experience stress related to their sexual orientation due to the cultural characteristics of Asian communities; they also experienced stress due to the COVID-19 pandemic. The COVID-19 pandemic amplified existing inequities related to sexual orientation. The Director-General of the World Health Organization announced on 3 May 2023, that COVID-19 was no longer an international public health emergency (WHO, 2023b). Nevertheless, health professionals must consider the mental health of LGB individuals and prevent suicide in this population.

The present study discovered that LGB individuals who were transgender, who perceived greater levels of sexual microaggression and who had greater problematic Internet use at baseline were more likely to have new-onset suicide risk at follow-up. LGB individuals who had greater levels of self-identity confusion were more likely to have persistent suicide risk at follow-up. Both LGB and transgender populations experience minority-related stress, which partially accounts for the high suicide risk in these two populations (de Lange *et al.*, 2022). Transgender LGB individuals may encounter dual minority-related stress and have a higher suicide risk than cisgender LGB individuals do. A meta-analysis demonstrated that gender nonconformity is associated with perceiving higher numbers of prejudicial events and having higher expectations of rejection related to sexual orientation (Thoma *et al.*, 2021). Although the number of transgender LGB individuals is small, mental health in this population must be taken care of.

The present study found that perceived sexual microaggression but not perceived familial or internalized sexual stigma predicted new-onset suicide risk among LGB individuals who experienced the COVID-19 pandemic. This study is the first to consider the multifaceted nature of perceived sexual stigma and to analyze the predictive effects of different types of sexual stigma on suicide risk in LGB individuals who experienced the COVID-19 pandemic. Sexual microaggression is a form of discrimination that stems from heterosexism and involves indignities presented in the forms of verbal expressions, behaviours and environments that convey slights or insults towards LGB individuals (Sue *et al.*, 2007; Swann *et al.*, 2016). Sexual microaggression is often subtle and unintentional. Nevertheless, sexual microaggression is significantly associated with depression, anxiety and suicide in LGB individuals (Nadal *et al.*, 2011; Tsai *et al.*, 2021). Enactors of sexual microaggression often view their behaviour as harmless and well intentioned, and targets of sexual microaggression may find it difficult to communicate their feelings about sexual microaggression to enactors (Nadal *et al.*, 2016). LGB individuals may feel disturbed about and wary of what people say and do, and this can increase the risks of mental health problems such as depression, anxiety, traumatic stress symptoms, alcohol use and abuse, cannabis use and problems and suicidal ideation and attempt (DeSon and Andover, 2024). Sexual orientation-based microaggressions are also the risk of internalized sexual stigma (DeSon and Andover, 2024). The overall negative impacts of microaggressions on LGB individuals may increase their difficulties in adapting to the status quo during the COVID-19 pandemic, thus increasing the risk of suicide. The findings of this study indicate that intervention programmes may be beneficial in reducing sexual microaggression in society and developing cognitive and emotional coping strategies among LGB individuals who perceive sexual microaggression.

Cross-sectional studies have discovered that perceived familial sexual stigma (de Lange *et al.*, 2022) and internalized sexual stigma (Lee *et al.*, 2019) are significantly associated with suicide in LGB individuals. However, the present prospective study did not find significantly predictive effects of familial or internalized sexual stigma on suicide risk among LGB individuals. Additional research is required to replicate these findings.

Cross-sectional studies have identified an association between problematic Internet use and suicide risk among young adults (Bersani *et al.*, 2022; Stevens *et al.*, 2020). Both problematic Internet use and suicide may result from various psychopathologies, such as mood disorders, impulsivity and aggression (Huang *et al.*, 2020). In addition, problematic Internet use may increase sleep disturbance (Guo *et al.*, 2018) and decrease quality of life (Huang *et al.*, 2020), leading to an increase in the risk of suicide. Although LGB individuals may use the Internet to confirm their sexual identity (Zhang *et al.*, 2022), connect with others in LGB communities (Berger *et al.*, 2022; Ybarra *et al.*, 2015) and find sexual partners (Ko *et al.*, 2016), problematic Internet use can limit their opportunities for real-world interactions and their access to sources of support, thereby potentially increasing their risk of suicide. The findings of the present study indicate that intervention programmes that treat problematic Internet use may reduce the risk of suicide among LGB individuals.

The present study revealed that LGB individuals with greater levels of self-identity confusion were more likely to have persistent suicide risk at follow-up. Individuals who have a firm sense of self-identity have social and emotional competence, self-efficacy and consistent beliefs and values across time and contexts (Erikson, 1968; Gemelli, 1996), whereas individuals who lack a firm sense of self-identity have an unstable self-image and unrealistic expectations regarding future goals, friends and sexual orientation identity (Kaufman *et al.*, 2015). Individuals without a firm sense of self-identity might have difficulty finding purpose in life, especially during times of social upheaval, such as the COVID-19 pandemic; these individuals might have greater difficulty in coping with these types of crises than those with a firmer sense of self-identity do.

Studies have reported that depression and anxiety are significantly associated with suicide in LGB individuals (Mustanski and Liu, 2013; Skerrett *et al.*, 2016; Tan *et al.*, 2021); however, in the present study, neither depression nor anxiety were significant predictors of suicide in LGB individuals. The present study considered the effect of sexual stigma and self-identity confusion that may contribute to depression and anxiety in LGB individuals and therefore influence the prediction of depression and anxiety on the risk of suicide. A previous study indicated that low family function predicted suicide in LGB individuals (Mustanski and Liu, 2013). Conflict with family members due to sexual orientation was identified as a stressor among LGB individuals during the COVID-19 pandemic (Suen *et al.*, 2020). However, in the present study, perceived family support was not a significant predictor of suicide in LGB individuals.

Notably, this study had several limitations. First, because we collected data from a single source, our results may have been subject to shared-method variance. Second, our participants were interested in the follow-up survey. Those who did not complete the follow-up survey were more likely to have an educational level of high school or lower. Thus, our results may not be generalizable to all LGB individuals. Third, several factors were not evaluated at baseline, including patient history of HIV and economic status, and thus the predictive effects of these factors on the new onset and persistent suicide risk remain undetermined. Fourth, the participants of this study were LGB individuals but not the individuals with a questioning sexual orientation. Questioning individuals, especially young individuals are at higher risk for bullying victimization, suicidal thoughts and drug and alcohol abuse more so than LGB individuals, possibly due to marginalization from straight and LGB peers alike (Hutchison, 2010). Whether the results of this study can be generalized to the individuals with a questioning sexual orientation warrants further study.

## Conclusions

Being transgender, perceiving greater levels of sexual microaggression, and having greater problematic Internet use at baseline predicted new-onset suicide risk at follow-up among LGB individuals; having greater levels of self-identity confusion at baseline predicted persistent suicide risk at follow-up. Therefore, efforts should be made to modify public attitudes towards LGB and transgender individuals and reduce the occurrence of sexual microaggression. Furthermore, healthcare providers are recommended to develop programmes that help LGB individuals develop a firm sense of self-identity and skills related to managing Internet usage. Mental health problems among LGB individuals should be considered a key health concern and should be further investigated in-depth.

## Data Availability

The data will be available upon reasonable request to the corresponding authors.

## References

[ref1] Batra K, Pharr JR, Kachen A, Godbey S and Terry E (2023) Investigating the psychosocial impact of COVID-19 among the sexual and gender minority population: A systematic review and meta-analysis. *LGBT Health* 10, 416–428. 37022764 10.1089/lgbt.2022.0249

[ref2] Berger M, Taba M, Marino J, Lim M and Skinner S (2022) Social media use and health and well-being of lesbian, gay, bisexual, transgender, and queer youth: Systematic review. *Journal of Medical Internet Research* 24, e38449. 10.2196/38449 PMC9536523 36129741

[ref3] Bersani FS, Accinni T, Carbone GA, Corazza O, Panno A, Prevete E, Bernabei L, Massullo C, Burkauskas J, Tarsitani L, Pasquini M, Biondi M, Farina B and Imperatori C (2022) Problematic use of the Internet mediates the association between reduced mentalization and suicidal ideation: A cross-sectional study in young adults. *Healthcare (Basel)* 10, 948. 10.3390/healthcare10050948 PMC9140488 35628085

[ref4] Broman N and Hakansson A (2018) Problematic gaming and internet use but not gambling may be overrepresented in sexual minorities - A pilot population web survey study. *Frontiers in Psychology* 9, 2184. 10.3389/fpsyg.2018.02184 PMC6243046 30483191

[ref5] Bronfenbrenner U (1979) *The Ecology of Human Development*. Cambridge, MA: Harvard University Press.

[ref6] Buspavanich P, Lech S, Lermer E, Fischer M, Berger M, Vilsmaier T, Kaltofen T, Keckstein S, Mahner S, Behr J, Thaler CJ and Batz F (2021) Well-being during COVID-19 pandemic: A comparison of individuals with minoritized sexual and gender identities and cis-heterosexual individuals. *PloS one* 16, e0252356. 10.1371/journal.pone.0252356 PMC8186787 34101746

[ref7] Chen C, Haupert SR, Zimmermann L, Shi X, Fritsche LG and Mukherjee B (2022) Global prevalence of post-coronavirus disease 2019 (COVID-19) condition or long COVID: A meta-analysis and systematic review. *Journal of Infectious Diseases* 226, 1593–1607. 35429399 10.1093/infdis/jiac136 PMC9047189

[ref8] Chen JH (2022) Disparities in mental health and well-being between heterosexual and sexual minority older adults during the COVID-19 pandemic. *Journal of Aging and Health* 34, 939–950. 35430925 10.1177/08982643221081965 PMC9014338

[ref9] Chen JS, Huang YT, Lin CY, Yen CF, Griffiths MD and Pakpour AH (2021) Relationships of sexual orientation microaggression with anxiety and depression among lesbian, gay, and bisexual Taiwanese youth: Self-identity disturbance mediates but gender does not moderate the relationships. *International Journal of Environmental Research & Public Health* 18, 12981. 10.3390/ijerph182412981 PMC8701819 34948591

[ref10] Chen S, Weng L, Su Y, Wu H and Yang P (2003) Development of Chinese Internet Addiction Scale and its psychometric study. *Chinese Journal of Psychology* 45, 279–294.

[ref11] Chen TH, Hsiao RC, Liu TL and Yen CF (2019) Predicting effects of borderline personality symptoms and self-concept and identity disturbances on internet addiction, depression, and suicidality in college students: A prospective study. *Kaohsiung Journal of Medical Sciences* 35, 508–514. 31063227 10.1002/kjm2.12082 PMC11900761

[ref12] Chen Y, Hsu C, Hsu S and Lin C (1980) A preliminary study of family APGAR index. *Acta Paediatrica Sinica* 21, 210–217.

[ref13] Chen YL, Chang YP and Yen CF (2023) Effects of gender nonconformity and biological sex on the relationship between sexual orientation microaggressions and anxiety and depressive symptoms among lesbian, gay, and bisexual Taiwanese young adults: A moderated-moderation study. *Journal of Affective Disorders* 334, 129–136. 37150223 10.1016/j.jad.2023.04.131

[ref14] Chin WY, Choi EP, Chan KT and Wong CK (2015) The psychometric properties of the Center for Epidemiologic Studies Depression Scale in Chinese primary care patients: Factor structure, construct validity, reliability, sensitivity, and responsiveness. *PLoS one* 10, e0135131. 10.1371/journal.pone.0135131 PMC4529142 26252739

[ref15] de Lange J, Baams L, van Bergen DD, Bos HMW and Bosker RJ (2022) Minority stress and suicidal ideation and suicide attempts among LGBT adolescents and young adults: A meta-analysis. *LGBT Health* 9, 222–237. 35319281 10.1089/lgbt.2021.0106

[ref16] DeSon JJ and Andover MS (2024) Microaggressions toward sexual and gender minority emerging adults: An updated systematic review of psychological correlates and outcomes and the role of intersectionality. LGBT *Health* 11, 249–268. 37906109 10.1089/lgbt.2023.0032

[ref17] Diaz A, Baweja R and Bonatakis JK (2021) Global health disparities in vulnerable populations of psychiatric patients during the COVID-19 pandemic. *World Journal of Psychiatry* 11, 94–108. 33889535 10.5498/wjp.v11.i4.94 PMC8040151

[ref18] Erikson E (1968) *Identity: Youth and Crisis*. New York: W.W. Norton & Company.

[ref19] Gemelli RJ (1996) *Normal Child and Adolescent Development*. Washington, DC: American Psychiatric Press.

[ref20] Gibb JK, DuBois LZ, Williams S, McKerracher L, Juster RP and Fields J (2020) Sexual and gender minority health vulnerabilities during the COVID-19 health crisis. *American Journal of Human Biology* 32, e23499. 10.1002/ajhb.23499 32910838

[ref21] Guo L, Luo M, Wang WX, Huang GL, Xu Y, Gao X, Lu CY and Zhang WH (2018) Association between problematic Internet use, sleep disturbance, and suicidal behavior in Chinese adolescents. *Journal of Behavioral Addictions* 7, 965–975. 30474380 10.1556/2006.7.2018.115 PMC6376369

[ref22] Hsieh MT, Chen JS, Lin CY, Yen CF, Griffiths MD and Huang YT (2021) Measurement invariance of the Sexual Orientation Microaggression Inventory across LGB males and females in Taiwan: Bifactor structure fits the best. *International Journal of Environmental Research & Public Health* 18, 10668. 10.3390/ijerph182010668 PMC8536138 34682410

[ref23] Huang MF, Chang YP, Lu WH and Yen CF (2022) Problematic smartphone use and its associations with sexual minority stressors, gender nonconformity, and mental health problems among young adult lesbian, gay, and bisexual individuals in Taiwan. *International Journal of Environmental Research & Public Health* 19, 5780. 10.3390/ijerph19095780 PMC9105429 35565175

[ref24] Huang Y, Xu L, Mei Y, Wei Z, Wen H and Liu D (2020) Problematic Internet use and the risk of suicide ideation in Chinese adolescents: A cross-sectional analysis. *Psychiatry Research* 290, 112963. 10.1016/j.psychres.2020.112963 32450410

[ref25] Hutchison ED (2010) *Dimensions of Human Behavior: The Changing Life Course*. New York: SAGE, 252.

[ref26] Kamal K, Li JJ, Hahm HC and Liu CH (2021) Psychiatric impacts of the COVID-19 global pandemic on U.S. sexual and gender minority young adults. *Psychiatry Research* 299, 113855. 10.1016/j.psychres.2021.113855 PMC8278978 33721788

[ref27] Kaufman EA, Cundiff JM and Crowell SE (2015) The development, factor structure, and validation of the Self-Concept and Identity Measure (SCIM): A self-report assessment of clinical identity disturbance. *Journal of Psychopathology and Behavioral Assessment* 37, 122–133.

[ref28] Kaufman EA, Puzia ME, Crowell SE and Price CJ (2019) Replication of the Self-Concept and Identity Measure (SCIM) among a treatment-seeking sample. *Identity* 19, 18–28. 31602176 10.1080/15283488.2019.1566068 PMC6786792

[ref29] Ko CH, Yen JY, Chen SH, Yang MJ, Lin HC and Yen CF (2009) Proposed diagnostic criteria and the screening and diagnosing tool of Internet addiction in college students. *Comprehensive Psychiatry* 50, 378–384. 19486737 10.1016/j.comppsych.2007.05.019

[ref30] Ko N, Tseng P, Huang Y, Chen Y and Hsu S (2016) Seeking sex partners through the internet and mobile phone applications among men who have sex with men in Taiwan. *AIDS Care* 28, 927–931. 26754350 10.1080/09540121.2015.1131969

[ref31] Krause KD (2021) Implications of the COVID-19 pandemic on LGBTQ communities. *Journal of Public Health Management and Practice* 27, S69–S71. 33239566 10.1097/PHH.0000000000001273

[ref32] Lee CH, Lai CL, Sung YH, Lai MY, Lin CY and Lin LY (2017) Comparing effects between music intervention and aromatherapy on anxiety of patients undergoing mechanical ventilation in the intensive care unit: A randomized controlled trial. *Quality of Life Research* 26, 1819–1829. 28236262 10.1007/s11136-017-1525-5

[ref33] Lee H, Operario D, Yi H, Choo S and Kim SS (2019) Internalized homophobia, depressive symptoms, and suicidal ideation among lesbian, gay, and bisexual adults in South Korea: An age-stratified analysis. *LGBT Health* 6, 393–399. 31746660 10.1089/lgbt.2019.0108

[ref34] Lee JI, Chang YP, Tsai CS and Yen CF (2022) Internalized sexual stigma among lesbian, gay, and bisexual individuals in Taiwan: Its related factors and association with mental health problems. *International Journal of Environmental Research & Public Health* 19, 2427. 10.3390/ijerph19042427 PMC8872568 35206614

[ref35] Lin CY, Griffiths MD, Pakpour AH, Tsai CS and Yen CF (2022) Relationships of familial sexual stigma and family support with internalized homonegativity among lesbian, gay and bisexual individuals: The mediating effect of self-identity disturbance and moderating effect of gender. *BMC Public Health* 22, 1465. 10.1186/s12889-022-13815-4 PMC9344633 35915488

[ref36] Lin CY, Luh WM, Cheng CP, Yang AL, Su CT and Ma HI (2013) Measurement equivalence across child self-reports and parent-proxy reports in the Chinese version of the pediatric quality of life inventory version 4.0. *Child Psychiatry & Human Development* 44, 583–590. 23242709 10.1007/s10578-012-0352-8

[ref37] Lingiardi V, Baiocco R and Nardelli N (2012) Measure of Internalized Sexual Stigma for Lesbians and Gay Men: A new scale. *Journal of Homosexuality* 59, 1191–1210. 22966998 10.1080/00918369.2012.712850

[ref38] Liu H, Feng T and Rhodes AG (2009) Assessment of the Chinese version of HIV and Homosexuality related stigma scales. *Sexually Transmitted Infections* 85, 65–69. 18790858 10.1136/sti.2008.032714

[ref39] Liu RT and Mustanski B (2012) Suicidal ideation and self-harm in lesbian, gay, bisexual, and transgender youth. *American Journal of Preventive Medicine* 42, 221–228. 22341158 10.1016/j.amepre.2011.10.023

[ref40] Lozano-Verduzco I, Vega-Cauich J, Mendoza-Pérez JC and Craig SL (2023) Perceived social support and mental health indicators of a Mexican LGBT sample during the COVID-19 pandemic. *International Journal of Ment Health and Addiction*. 10.1007/s11469-023-01064-4 PMC10153024 37363771

[ref41] Miranda-Mendizábal A, Castellví P, Parés-Badell O, Almenara J, Alonso I, Blasco MJ, Cebrià A, Gabilondo A, Gili M, Lagares C, Piqueras JA, Roca M, Rodríguez-Marín J, Rodríguez-Jiménez T, Soto-Sanz V, Vilagut G and Alonso J (2017) Sexual orientation and suicidal behaviour in adolescents and young adults: Systematic review and meta-analysis. *British Journal of Psychiatry* 211, 77–87. 10.1192/bjp.bp.116.196345 28254960

[ref42] Mustanski B and Liu RT (2013) A longitudinal study of predictors of suicide attempts among lesbian, gay, bisexual, and transgender youth. *Archives of Sexual Behavior* 42, 437–448. 23054258 10.1007/s10508-012-0013-9

[ref43] Nadal KL, Whitman CN, Davis LS, Erazo T and Davidoff KC (2016) Microaggressions toward lesbian, gay, bisexual, transgender, queer, and genderqueer people: A review of the literature. *Journal of Sex Research* 53, 488–508. 26966779 10.1080/00224499.2016.1142495

[ref44] Nadal KL, Wong Y, Issa MA, Meterko V, Leon J and Wideman M (2011) Sexual orientation microaggressions: Processes and coping mechanisms for lesbian, gay, and bisexual individuals. *Journal of LGBT Issues in Counseling* 5, 21–46.

[ref45] Onyeaka H, Anumudu CK, Al-Sharify ZT, Egele-Godswill E and Mbaegbu P (2021) COVID-19 pandemic: A review of the global lockdown and its far-reaching effects. *Science Progress* 104, 368504211019854. 10.1177/00368504211019854 PMC10454957 34061685

[ref46] Radloff LS (1977) The CES-D scale: A self-report depression scale for research in the general population. *Applied Psychological Measurement* 1, 385–401.

[ref47] Salerno JP, Williams ND and Gattamorta KA (2020) LGBTQ populations: Psychologically vulnerable communities in the COVID-19 pandemic. *Psychological Trauma* 12, S239–S242. 32551761 10.1037/tra0000837 PMC8093609

[ref48] Sheehan DV, Lecrubier Y, Sheehan KH, Amorim P, Janavs J, Weiller E, Hergueta T, Baker R and Dunbar GC (1998) The Mini-International Neuropsychiatric Interview (MINI): The development and validation of a structured diagnostic psychiatric interview for DSM-IV and ICD-10. *Journal of Clinical Psychiatry* 59, 22–33. 9881538

[ref49] Skerrett DM, Kõlves K and De Leo D (2016) Factors related to suicide in LGBT populations. *Crisis* 37, 361–369. 27659515 10.1027/0227-5910/a000423

[ref50] Slemon A, Richardson C, Goodyear T, Salway T, Gadermann A, Oliffe JL, Knight R, Dhari S and Jenkins EK (2022) Widening mental health and substance use inequities among sexual and gender minority populations: Findings from a repeated cross-sectional monitoring survey during the COVID-19 pandemic in Canada. *Psychiatry Resesrach* 307, 114327. 10.1016/j.psychres.2021.114327 PMC8647565 34923446

[ref51] Smilkstein G (1978) The family APGAR: A proposal for a family function test and its use by physicians. *Journal of Family Practice* 6, 1231–1239. 660126

[ref52] Spielberger CD, Gorsuch RL, Lushene R, Vagg PR and Jacobs GA (1983) *Manual for the State-Trait Anxiety Inventory*. Palo Alto, CA: Consulting Psychologists Press.

[ref53] Stevens C, Zhang E, Cherkerzian S, Chen JA and Liu CH (2020) Problematic internet use/computer gaming among US college students: Prevalence and correlates with mental health symptoms. *Depression and Anxiety* 37, 1127–1136. 32939888 10.1002/da.23094 PMC8635392

[ref54] Sue DW, Bucceri J, Lin AI, Nadal KL and Torino GC (2007) Racial microaggressions and the Asian American experience. *Cultural Diversity and Ethnic Minority Psychology* 13, 72–81. 17227179 10.1037/1099-9809.13.1.72

[ref55] Suen YT, Chan R and Wong E (2020) Effects of general and sexual minority-specific COVID-19-related stressors on the mental health of lesbian, gay, and bisexual people in Hong Kong. *Psychiatry Research* 292, 113365. 10.1016/j.psychres.2020.113365 PMC7397990 32862107

[ref56] Swann G, Minshew R, Newcomb ME and Mustanski B (2016) Validation of the Sexual Orientation Microaggression Inventory in two diverse samples of LGBTQ youth. *Archives of Sexual Behavior* 45, 1289–1298. 27067241 10.1007/s10508-016-0718-2 PMC4945424

[ref57] Tan RKJ, Low TQY, Le D, Tan A, Tyler A, Tan C, Kwok C, Banerjee S, Cook AR and Wong ML (2021) Experienced homophobia and suicide among young gay, bisexual, transgender, and queer men in Singapore: Exploring the mediating role of depression severity, self-esteem, and outness in the Pink Carpet Y Cohort Study. *LGBT Health* 8, 349–358. 34142861 10.1089/lgbt.2020.0323 PMC8252908

[ref58] Thoma BC, Eckstrand KL, Montano GT, Rezeppa TL and Marshal MP (2021) Gender nonconformity and minority stress among lesbian, gay, and bisexual individuals: A meta-analytic review. *Perspectives on Psychological Science* 16, 1165–1183. 33645322 10.1177/1745691620968766 PMC7646043

[ref59] Tsai CS, Huang YT and Yen CF (2021) Experience of sexual orientation microaggression among young adult lesbian, gay, and bisexual individuals in Taiwan: Its related factors and association with mental health problems. *International Journal of Environmental Research & Public Health* 18, 11744. 10.3390/ijerph182211744 PMC8624001 34831500

[ref60] Veldhuis CB, Nesoff ED, McKowen ALW, Rice DR, Ghoneima H, Wootton AR, Papautsky EL, Arigo D, Goldberg S and Anderson JC (2020) Addressing the critical need for long-term mental health data during the COVID-19 pandemic: Changes in mental health from April to September 2020. *Preventive Medicine* 146, 106465. 10.1016/j.ypmed.2021.106465 PMC8136863 33647353

[ref61] Wang CC, Chang YP, Yang YH, Hu HF and Yen CF (2019) Relationships between traditional and cyber harassment and self-identity confusion among Taiwanese gay and bisexual men in emerging adulthood. *Comprehensive Psychiatry* 90, 14–20. 30639893 10.1016/j.comppsych.2018.12.015

[ref62] World Health Organization, 2023a. WHO Coronavirus (COVID-19) Dashboard. https://covid19.who.int/ (accessed 2 April 2024).

[ref63] World Health Organization, 2023b. Statement on the fifteenth meeting of the International Health Regulations. Emergency Committee regarding the coronavirus disease (COVID-19) pandemic. URL: https://www.who.int/news/item/05-05-2023-statement-on-the-fifteenth-meeting-of-the-international-health-regulations-(2005)-emergency-committee-regarding-the-coronavirus-disease-(covid-19)-pandemic (accessed 2 April 2024).

[ref64] Ybarra M, Mitchell K, Palmer N and Reisner S (2015) Online social support as a buffer against online and offline peer and sexual victimization among U.S. LGBT and non-LGBT youth. *Child Abuse and Neglect* 39, 123–136. 25192961 10.1016/j.chiabu.2014.08.006 PMC6483382

[ref65] Yen CF, Huang YT, Potenza MN, Tsai TT, Lin CY and Tsang HWH (2021) Measure of Internalized Sexual Stigma for Lesbians and Gay Men (MISS-LG) in Taiwan: Psychometric evidence from Rasch and confirmatory factor analysis. *International Journal of Environmental Research & Public Health* 18, 13352. 10.3390/ijerph182413352 PMC8703518 34948970

[ref66] Zhang A, Reynolds N, Huang C, Tan S, Yang G and Yan J (2022) The process of contemporary gay identity development in China: The influence of internet use. *Frontiers in Public Health* 10, 954674. 10.3389/fpubh.2022.954674 PMC9486466 36148342

